# Role of Micronutrients and Gut Microbiota-Derived Metabolites in COVID-19 Recovery

**DOI:** 10.3390/ijms232012324

**Published:** 2022-10-14

**Authors:** Teresita de Jesús Hernández-Flores, Eliza Julia Pedraza-Brindis, Jhonathan Cárdenas-Bedoya, José Daniel Ruíz-Carrillo, Anibal Samael Méndez-Clemente, Marco Alonso Martínez-Guzmán, Liliana Iñiguez-Gutiérrez

**Affiliations:** 1 Departamento de Disciplinas Filosófico, Metodológicas e Instrumentales, Centro Universitario de Ciencias de la Salud, Universidad de Guadalajara, Guadalajara 44340, Jalisco, Mexico; 2 Instituto de Investigación de Inmunodeficiencias y VIH, Hospital Civil de Guadalajara “Fray Antonio Alcalde”, Guadalajara 44280, Jalisco, Mexico; 3 Departamento de Aparatos y Sistemas I, Facultad de Medicina, Universidad Autónoma de Guadalajara, Guadalajara 44670, Jalisco, Mexico; 4 Laboratorio de Inmunodeficiencias y Retrovirus Humanos, Centro de Investigación Biomédica de Occidente, Centro Médico Nacional de Occidente, Instituto Mexicano del Seguro Social, Guadalajara 44340, Jalisco, Mexico; 5 Clínica Medicina Familiar 1 del ISSSTE “Dr. Arturo González Guzmán”, Guadalajara 44340, Jalisco, Mexico

**Keywords:** gut microbiota, SARS-CoV-2, COVID-19, COVID-19 recovery, microbiota-derived metabolites, micronutrients

## Abstract

A balanced and varied diet provides diverse beneficial effects on health, such as adequate micronutrient availability and a gut microbiome in homeostasis. Besides their participation in biochemical processes as cofactors and coenzymes, vitamins and minerals have an immunoregulatory function; meanwhile, gut microbiota and its metabolites coordinate directly and indirectly the cell response through the interaction with the host receptors. Malnourishment is a crucial risk factor for several pathologies, and its involvement during the Coronavirus Disease 2019 pandemic has been reported. This pandemic has caused a significant decline in the worldwide population, especially those with chronic diseases, reduced physical activity, and elder age. Diet and gut microbiota composition are probable causes for this susceptibility, and its supplementation can play a role in reestablishing microbial homeostasis and improving immunity response against Coronavirus Disease 2019 infection and recovery. This study reviews the role of micronutrients and microbiomes in the risk of infection, the severity of disease, and the Coronavirus Disease 2019 sequelae.

## 1. Introduction

The Coronavirus Disease 2019 (COVID-19) is caused by the Severe Acute Respiratory Syndrome Coronavirus 2 (SARS-CoV-2) [1]. The disease is mainly characterized by inflammation and necrosis of lung tissue, although the virus has proved a surprisingly high pleiotropism. The target cells so far reported include pneumocytes, lung parenchyma epithelial cells, airways, and cells from the blood vessels, small intestine, central nervous system, kidney, and liver, where *angiotensin II converting enzyme ACE2* gene codes for ACE2 cell receptor (SARS-CoV-2 binding receptor) in a wide variety of human tissues. Therefore, individuals may experience different symptoms due to SARS-CoV-2 infection. In addition, there is a high chance of sequels after recovery that manifest in the respiratory system and other organs that may have been infected [2]. Some groups are particularly vulnerable during the pandemic, especially those older than 60, individuals with reduced physical activity, and those with chronic degenerative diseases such as type II diabetes (T2D), cardiovascular disease (CVD), overweight, and obesity [3]. Most people with these conditions have poor nutrition and deteriorated immunity [4]. Remarkably, one out of three senior adults in developed countries has nutritional deficiencies due to sensory or mental issues, systemic chronic diseases, extreme poverty, low variability in diets, poor absorption, polypharmacy, and other related factors [4,5]. Several international institutions state that “no foods or dietary supplements can prevent or cure COVID-19 infection”; however, the nutritional state plays a vital role in the health of individuals, especially regarding their ability to overcome infections since the timely onset of an immune response is dependent on the nutrition [6]. In addition, the probability of developing into a severe stage of COVID-19 increases significantly in adults with malnutrition, according to a scientific report that included 102,099 hospitalized patients in the United States of America (USA) [7]. It is worth mentioning that malnutrition is a condition generally accepted as an insufficient amount of calories, but other vital nutrients, such as vitamins and minerals, are just as crucial for good health. Even individuals with overweight or with obesity are at risk of malnutrition derived from a lack of micronutrients [8]. The European Food Safety Authority (EFSA) highlighted the importance of micronutrients for the proper functioning of the immune system, which remarks on the role of Vitamins D, A, B6, B9, B12, and C, as well as the minerals such as iron (Fe), copper (Cu), selenium (Se), and zinc (Zn) [9].

The interest of healthcare professionals is to promote the ingestion of varied foods rich in nutrients to prevent micronutrient deficiency. Furthermore, a changing diet is a key “modifiable” factor capable of inducing changes in gut microbiota (GM). This microbiota is composed of a diverse group of viruses, fungi, parasites, and bacteria; of these microorganisms, bacteria are of great interest because 99.1% of the genes in the catalog of the GM are of bacterial origin [10], according to the proportion and quantity of microorganisms, a different type and load of metabolic products will be obtained. Therefore, an adequate food intake can modulate GM composition and its metabolites in COVID-19 infection and reestablish immunity response to infections [11]. Current nutritional research perspectives include studying the molecular mechanisms involved in nutrients’ impact on the immune system and the levels of interaction between the microbiome and the human organism, such as genomic, epigenetic, cellular, tissular, organic, and systemic.

In this work, we will review the role of micronutrients, microbiota, and their metabolites in the process of infection and recovery from COVID-19.

## 2. Role of Micronutrients in Individual Health and Disease

**Figure 1:**
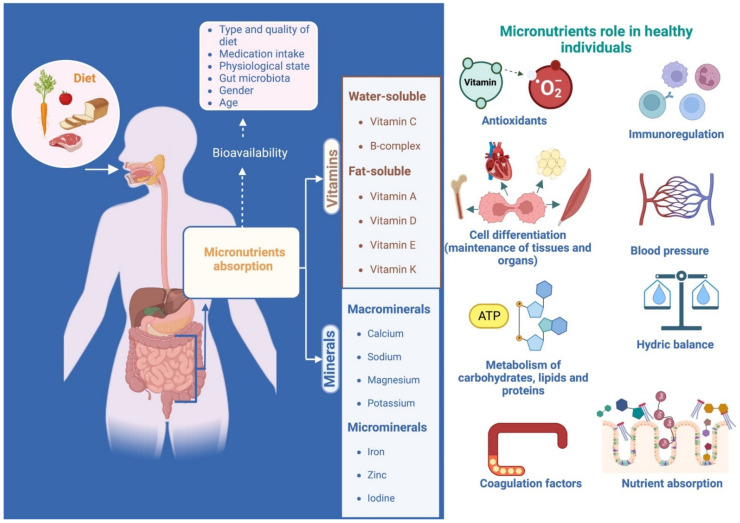
Micronutrients role in healthy individuals. Whether micronutrients’ daily intake requirements are grams or as much as micrograms, their presence is critical to several body systems regulations. Their stand-out tasks are oxidation-reduction process modulation, immunoregulation, cell differentiation, water and electrolyte balance, homeostasis in arterial pressure, well-functioning coagulation factors, and the synthesis and absorption of some macronutrients and micronutrients. Its bioavailability, absorption, and synthesis rely on factors such as age, gender, diet, GM, pharmaceutical prescriptions, and pre-existing metabolic or pathological conditions (created with BioRender.com).

Micronutrients are elements classified as vitamins and minerals. Optimal concentrations can function as co-factors and coenzymes and participate in metabolic activity in biochemical processes. Its concentrations depend on the metabolic activity and the life cycle stage, as well as specific dietary habits and infectious processes [12]. An inappropriate pool of micronutrients in the individual may lead to a deficiency, increasing the risk of certain diseases and may lead to other micronutrient deficiencies. This nutritional lack may go unnoticed, and the signs and symptoms usually appear only in the advanced stages of the disease, such as anemia, pellagra, beriberi, and others [13]. Figure 1 spotlights the actions of micronutrients carried out in the human body when they are balanced and in adequate quantities.

Vitamins are micronutrients with particular physical-chemical features; classified as fat-soluble and water-soluble, which include vitamins A, C, D, and K, and vitamins of the B complex: thiamin (B1), riboflavin (B2), niacin (B3), pantothenic acid (B5), pyridoxine (B6), biotin (B7), folic acid (B9), and cobalamin (B12). These vitamins have diverse functions depending on the cell target [14,15]. On the other hand, minerals are inorganic elements in the soil or water, essential for organisms but highly toxic in excess. These elements are critical for the body’s construction and functioning of biomolecules. Even though they do not provide energy, they are necessary to maintain biochemical processes in the body. Based on human requirements, there are two types: macrominerals (calcium (Ca), sodium (Na), magnesium (Mg), potassium (K), and phosphorus (P)) and microminerals (trace elements such as Fe, chlorine (Cl), cobalt (Co), Cu, Zn, iodine (I), and (Se) (Table 1) [14,16,17,18,19].

### 2.1. Water-Soluble Vitamins

Vitamin B is a cofactor in diverse cellular reactions and mediates amino acid synthesis. Within this group are vitamins B2, B3, B6, and B12, essential in the immune system against infection response; moreover, vitamin B2 is involved in the metabolic processes of cellular energy production [18].

Vitamin C or ascorbic acid relies on its potent antioxidant capacity, which regulates stress and oxidative damage in the body by controlling reactive oxygen species (ROS) acting as an electron donor [20]. At the immune system level, vitamin C and Zn are responsible for regulating cell division and adequate proliferative response [21].

### 2.2. Fat-Soluble Vitamins

Vitamin A, or retinoic acid, acts as a hormone at the systemic level and regulates Type-I interferons (IFN) synthesis in the immune system through nuclear retinoic acid receptors (RAR and RXR) signaling. Also, it is responsible for permanent immune system responses to viral infections through the Retinoic-acid-inducible gene I (RIG-I) signaling pathway. Additionally regulates Nuclear factor κB (NF-kB) activation [22,23]. Vitamin D improves innate immunity through cathelicidins and defensins synthesis by neutrophils and macrophages. These actions control viral replication and downregulate Tumor necrosis factor (TNF) alpha and IFN gamma expression [24,25]. Low levels of vitamin D (<20 ng/mL) are related to the development and progression of chronic diseases such as CVD, T2D, cancer, and depression, and also to poor bone health [26,27]. Vitamin E can affect immune system cells because of its antioxidant activity, protein kinase C (PKC) inhibition, and signal transduction through enzymatic modulation. In macrophages, vitamin E modifies cyclooxygenase activity, which controls peroxynitrite synthesis. This results in lower prostaglandin E2 production and upregulated T lymphocyte response that is T cell-mediated. Additionally, it improves Natural Killer (NK) cell activity through nitric oxide regulation [28]. Vitamin K is critical for synthesizing some clotting factors and blood homeostasis for chronic, low-inflammatory diseases such as CVD, osteoarthritis, dementia, and cognitive impairment, among others. It can naturally be found as phylloquinone (vitamin K1) and menaquinones (vitamin K2), which differ in source, absorption rate, tissue distribution, bioavailability, and target activity [29,30].

### 2.3. Macrominerals

Calcium is an essential element in bone mineralization and recently was related to influencing the wound healing process. Together with Vitamin D and adequate water intake (limited mineral water consumption), it allows excellent bioavailability, developing its optimal functions [31,32,33]. Sodium is a critical mineral in maintaining appropriate blood volume and pressure. It regulates the expression of the β-myosin heavy chain, α/β-myosin heavy chain, and myocyte enhancer factor 2/nuclear factor of activated T cell transcriptional activity [34]. Magnesium acts as a cofactor in the enzymatic reactions in the liver and kidneys; it blocks painful stimuli by sensory neurons, an antagonist action at the N-methyl-d-aspartate (NMDA) receptor. It is also a mediator of the physiological stress response, and its balance in the body help in the prevention of oxidative stress and chronic conditions associated with aging; together with vitamin D, it regulates calcium and phosphate homeostasis to influence the growth and maintenance of bones. Therefore, alleviates fibromyalgia, dysmenorrhea, headaches, and acute migraine attacks. In a vicious cycle, it limits its intestinal absorption and allows renal wasting when it is deficient. It can also induce skeletal deformities, CVD, and metabolic syndrome, including high blood pressure (HBP) and stroke, cardiometabolic syndrome and T2D, airway constrictive syndromes and asthma, and age-related symptoms such as asthenia, sleep disorders, hyperemotionality, and cognitive disorders [35,36,37,38]. Potassium, the principal cation inside cells, maintains cellular osmolarity and acid–base equilibrium, nerve stimulation transmission, and regulates cardiac and muscle functions. It is key to blood pressure homeostasis, being a regulator agent of ACE2 inhibitors and angiotensin I, promoting sodium excretion. It is also beneficial for conditions such as heart failure and chronic kidney disease, independent of their effect on blood pressure [39,40,41]. Phosphorus plays an important role, principally when it is in homeostasis with calcium and vitamin D, in maintaining bone health by directly becoming part of the bone mineral matrix affecting osteoblast and osteoclast proliferation. Lack of this homeostasis also increases the risk of infection, T2D, CVD, obesity, asthma, inflammatory bowel disease, colon, breast, prostate, and ovarian cancer, and some neurological diseases [42,43].

### 2.4. Microminerals

Zinc is the second most abundant mineral in the human body. It acts as a retinol cofactor in the immune system. Its availability in the organism significantly regulates the survival, differentiation, and proliferation of innate and adaptive immune systems; therefore, its deficiency seriously compromises the immune response of patients [44]. Zn can be free or linked to human and viral metalloenzymes. Supplementing this oligo element (and its ionophores) can help destabilize viral metalloenzymes. ACE2 is a metallopeptidase and the virus’s principal entrance pathway to the human host [45]. Fe is essential, but when in excess, potentially toxic micronutrient for all living cells. It is tightly controlled at cellular and systemic levels to prevent both deficiency and overload. Fe regulates post-transcriptionally the genes encoding proteins that modulate Fe uptake, recycling, and storage. The liver peptide hepcidin controls serum Fe through the degradation of ferroportin in enterocytes and macrophages. Also, the immune system requires Fe to sustain its function, metabolism, and proliferation, both invading pathogens and mammalian [46,47].

Micronutrients participate from initial interaction with pathogen–host to activation of the immune system [48], which requires interaction among micronutrients (vitamins A, C, D, E, B6, B12, B9) and trace minerals (such as Fe, Zn, Cu, Se, and Mg), acting as regulators of innate immune cell. They also produce pro- and anti-inflammatory cytokines, response to inflammation, oxidative burst function, reductive hemodynamic, T-cell differentiation, proliferation, interaction with viral antigens, and antibody production (Table 2) [16].

### 2.5. Water and Electrolyte Homeostasis

Water and electrolyte homeostasis mainly focuses on fluid body regulation, secretion, or absorption of minerals, which regulates water uptake and excretion by tissues and organs, where the kidney is the organ responsible for electrolyte and fluid status [49].

When fluid and electrolytes are in an electroneutrality state, they have an essential role in mechanisms that protect cell pH and produce normal gastrointestinal and renal secretions. Moreover, impacts physical performance and health in exercise, epithelial transport, history of physiology, hypoxia, and muscle performance, among others. Also have an essential role in the cardiovascular, central nervous, endocrinology, respiratory and gastrointestinal systems [50].

Sodium is the primary electrolyte in the extracellular compartment, with a serum range of 134–145 mmol/L. The Na+-K+ ATPase pump processes this mineral [51], allowing it to regulate blood volume, blood pressure, acid–base balance, and osmosis processes, and it is also a regulator of nerve and muscle function [52]. Potassium is a cation located at 98% in the intracellular fluid, and only 2% remains outside it [53]. With a serum range of 3.6–5.5 mmol/L, it is involved in several physiological mechanisms, such as insulin secretion by binding to specific cell receptors, causing glucose insertion to transporter type 4 (GLUT4), which facilitates glucose absorption in insulin-dependant tissues [54]. Calcium is present in the extracellular fluid with a range of 8.8–10.7 mmol/L, usually remaining 99% at the skeleton [55], while magnesium mainly settles the intracellular compartment with a range of 1.46–2.68 mg/dl and is mainly concentrated in the mitochondria. Together and in balance, these electrolytes are essential in regulating QT and JT intervals [56].

On the other hand, acid–base disturbances typically result in diseases such as HBP, cardiomyopathy, heart failure, arrhythmias, and even imbalances in the coagulation cascade [53,57]. Sodium is also the most prevalent electrolyte disturbance in hospitalized patients [52]. A high intake of sodium through the diet increases extracellular volume and cardiac output, leading to increased blood pressure and the risk of left ventricular hypertrophy, endothelial dysfunction, vascular remodeling, and kidney disease [58]. Furthermore, low calcium and high magnesium prolong QT and JT intervals, causing ventricular repolarization, which is associated with an increased risk of CVD and mortality [56].

## 3. Role of Micronutrients in COVID-19 Infection and Post-COVID-19 Syndrome Recovery

COVID-19 is an infectious disease caused by SARS-CoV-2 virus, characterized by a wide range of severity of respiratory symptoms, and in most severe cases, it can progress to pneumonia and systemic organ failure leading to death. According to the World Health Organization, around the world, 617,597,680 confirmed cases of COVID-19 have been reported, and of them, 6,532,705 resulted in death [59]. At the beginning of the COVID-19 pandemic, clinical features were dry cough, fever, fatigue, sore throat, headache, muscle pain, sore eyes, anosmia, ageusia, diarrhea, and difficulty breathing. Signs and symptoms remained for about 5–14 days even though some symptoms (cough, fatigue, dyspnea, myalgia, joint pain, and confusion) remained for weeks, up to three months after active SARS-CoV-2 infection [59,60,61,62]. Recent studies investigating the long-term effects of COVID-19 have found that a significant portion of patients continue to experience symptoms even after their acute infection has resolved. These post-COVID-19 effects can include fatigue, shortness of breath, and difficulties with cognition and concentration. While the severity of these symptoms varies from person to person, they can significantly impact the quality of life [63]. Sudre and collaborators with the COVID Symptom Study app found that those patients who presented more than five symptoms during the first weeks of the disease were associated with long COVID or post-COVID sequelae, in which symptoms are present for more than four weeks [64].

People with chronic diseases such as T2D, HBP, CVD, and older adults stage are susceptible to developing severe COVID-19, which is also related to a slow recovery [65]. During acute infection by COVID-19, a cytokine storm occurs as a response of the immune system to combat SARS-CoV-2 infection, which initiates an inflammatory state, that translates into high-demand energy or a hypermetabolic state [66]. Additional symptoms, such as gastrointestinal damage, anxiety, loss of appetite, and reduced physical condition, impact the patient’s nutritional status [67]. Therefore, physical activity, quality diet, and immune status factors influence COVID-19 and its sequelae recovery, which delays in patients whose nutrition has not been adequate [21].

Micronutrient deficiency suppresses the immune system by altering the T cells and the immune response mediated by antibodies. Vitamins in respiratory disease reduce cellular load and expression of viral antigens, diminish the expression of interferon regulatory factor 3 (*IRF3*) and mitochondrial antiviral signaling (*MAVS*) genes, and increase the expression of NF-κB. On the other hand, Sufficient intake of vitamins regulates both immune responses and, in specific cases, protects against the risk of infections by blocking the activity of pro-inflammatory cytokines such as TNF-α. Moreover, Interleukin 6 (IL-6) and IFNγ can reduce the incidence, severity, and risk of death from infections such as COVID-19 [19]. The immune system’s timely response to this infection determines the severity of the disease; therefore, micronutrients as immunoregulators are related to the response to COVID-19 infection [17]. Trace elements, such as Zn, Fe, and Se, are essential as protein cofactors that carry out activities such as immunoregulatory agents, cytoprotection, and growth factors. They also have anti-inflammatory, antiapoptotic, antiviral, and antibacterial activity [18,19]. Recent studies evaluate the nutritional status and the effect of dietary supplements on people during post-COVID-19 recovery. Micronutrient supplements that positively affect respiratory diseases, sepsis, and even COVID-19 are vitamins D, C, and B, and some minerals such as Zn [27]. Therefore, an approach to nutritional status and a specific diet to combat post-COVID-19 micronutrient deficiencies, such as vitamin D and Se, becomes necessary in these patients’ management [68].

### 3.1. Water-Soluble Vitamins

Serum concentrations of vitamin C can rapidly decrease depending on the body’s demand (e.g., infections). Its supplementation in patients decreases susceptibility to respiratory tract infections produced by COVID-19, and high intravenous doses (>24 g/dL daily) reduce the severity. Since ascorbic acid can inhibit the ACE2 expression in small alveolar epithelial cells, it is a form of regulating the entry of the SARS-CoV-2 into its target cells [20,69,70]. In patients with COVID-19, symptoms such as fatigue, pain, cognitive disorders, and depressive symptoms are linked to vitamin C deficiency. The same symptoms correlate with the most commonly present post-COVID syndrome. Its intravenous supplementation improves, reduces, and alleviates COVID-19 and post-COVID-19 symptoms [71,72]; however, more studies are needed to define the specific benefits and doses.

In silico analysis carried out by Wu et al. [73] predicted that B2 acts as an inhibitor of SARS-CoV-2 replication through the inhibition of PLpro (Papain-Like proteinase). This protein is essential for the N-terminal cleavage of the viral replicase polyprotein; a critical step for the subsequent release of factors (Nsp1, Nsp2, and Nsp3) required in viral replication. Vitamin B12, or methylcobalamin, has an affinity for the Nsp12 active site of SARS-CoV-2, which could cause inhibition of Nsp12 polymerase activity. In a study by Tan et al. [74] combined administration of vitamin B12, vitamin D, and magnesium in older COVID-19 patients, reduced the clinical deterioration prevalence, oxygen support need, and intensive care occupancy. The beneficial effects of vitamin B on post-COVID-19 syndrome also has been evaluated. Naureen et al. [61] selected a group of 20 people with post-COVID-19 syndrome (chronic fatigue as the primary symptom). Patients were supplemented daily with hydroxytyrosol, acetyl L-carnitine, and vitamins B, C, and D for 15 days; at the end of supplementation, they had almost doubled increase its energy levels and reduce fatigue and tiredness.

### 3.2. Fat-Soluble Vitamins

Like vitamin C, retinoic acid pool storage is rapidly consumed in response to viral load, resulting in an increase in fever [22,23]. Despite reports about the excellent regulation vitamin A has on the immune system and viral replication, there are no studies of its effect in COVID-19 or post-COVID syndrome patients.

Vitamin D deficiency allows for the survival and replication SARS-CoV-2 virus in the host. In addition, severe COVID-19 symptoms correlated with the most significant vitamin D deficiencies compared to mild COVID-19 [75,76]. This vitamin’s recommended daily intake doses are superior to 400 IU in patients with its depletion, particularly those hospitalized or with low sun exposure [77]. Recent works describe vitamin D deficiency affects cytokine synthesis, such as IL-6, IL-8, IL-12, TNF alpha, and IFN-gamma, altering cytokine storm in severe COVID-19 patients [78]. Although a few studies have studied vitamin D supplementation in COVID-19 patients, the authors report a mean serum concentration of 25-hydroxyvitamin D of 23–35 ng/mL. Biomarkers associated with the post-COVID-19 syndrome, such as D-dimer, IL-6, C-reactive protein, procalcitonin, and neutrophil count, did not show variations [25]. Only Gönen et al. [79] studied 95 hospitalized COVID-19 patients in Turkey, supplemented them with 25-hydroxyvitamin D, and showed a decrease in fibrinogen concentrations and vitamin D supplementation reduced hospital stays even in the presence of comorbidities.

Vitamin E effectively controls reactive species during active SARS-CoV-2 infection, which could be an essential regulator in this condition. Nevertheless, in ferroptosis process can be helpful, a common condition in COVID-19 patients. This Fe-dependent programmed cell death plays a vital role in multiple systemic diseases and damages the nervous system, lung, kidney, heart, liver, and gut, associated with the intracellular Fe and glutathione levels as if glutathione peroxidase 4 (GPX4) function capacity. COVID-19 patients suffer from GPX4 depletion, confirming ferroptosis; the principal mechanism behind the anti-ferroptosis effect of vitamin E is that it can detoxify oxidized lipids compensating for the lack of detoxification derived from GPX4 deficiency. It is worth mentioning that alpha-tocopherol hydroquinone is a more potent antioxidant than alpha-tocopherol [80]. Despite this, no studies have defined its role in the post-COVID-19 syndrome.

### 3.3. Minerals

About microminerals, till now, only one study has been published talking about their effect on COVID-19 and post-COVID-19 syndrome patients. The study, reported by Finci et al. [81] in 2020, showed that high doses of Zn salts (<200 mg daily) in COVID-19 patients improved oxygenation and decreased fever 24 h after treatment. Despite the small number of cases evaluated, this work establishes the possible beneficial effects of Zn in the viral control of SARS-CoV-2 and complications in COVID-19.

Additionally, for micronutrient management in post-COVID-19 patients, it is vital to maintain adequate hydration during the recovery phase, with a daily fluid intake of 2.5–3 L [77].

### 3.4. Water and Electrolyte Imbalance

The most severe patients affected by COVID-19 often present dehydration [53], diarrhea, and vomiting [82], which are potential factors for fluid distribution and electrolyte imbalance. Moreover, the main cardiovascular symptoms experienced by COVID-19 patients are heart palpitations and chest pain [83]. Some cardiovascular risk factors are also prevalent in hospitalized COVID-19 individuals, such as HBP at 56.6%, T2D at 33.8%, and acute myocardial injury at 12% [84,85,86].

Due to the high prevalence of CVD with COVID-19, researchers found that both conditions are strongly related [53]. The relationship might be due to SARS-CoV-2’s main entrance to the human host being through the ACE2 receptor, which participates in the renin-angiotensin-aldosterone system (RAAS). Moreover, RAAS is one of the principal causes of heart failure [87]. Therefore it is reasonable to admit that COVID-19 exacerbates and triggers early-onset CVD; likewise, patients with previous CVD may develop a more severe form of COVID-19; both scenarios significantly impact their post-COVID-19 syndrome recovery [53].

Lack of fluid electroneutrality, such as hypokalemia, hypomagnesemia, hyponatremia, and hypocalcemia, are pathophysiological alterations shared among CVD and COVID-19 [88]. Low sodium levels relate to an increased risk of mechanical ventilation in COVID-19 patients [89] and cause an inappropriate antidiuretic hormone secretion syndrome (SIADH), which is common in atypical viral pneumonia, such as SARS-CoV-2 infection [90]. Magnesium deficiency increases the incidence of CVD [91] and, in patients hospitalized with COVID-19, appears to be significantly correlated to patients’ unfavorable prognosis, especially those with a severe form of infection [92]. Alamdari et al. [93] demonstrated that hospital-admitted patients with this mineral deficiency have a higher mortality risk due to COVID-19. On the other hand, lower levels of serum K in confirmed cases of COVID-19, compared with non-infected patients [94,95], have more extended hospitalization and Intensive Care Unit (ICU) permanence, which represents an independent predictor of invasive mechanical ventilation [96]. In a retrospective study, only 51% of patients survived hospital discharge; almost 18% of all individuals developed a new-onset arrhythmia, and of them, 43% were mechanically ventilated [97]. A plausible mechanism linking COVID-19 to hypokalemia could be related to ACE2 receptor degradation after the virus has entered host cells [98]. Likewise, serum calcium imbalance as electrolyte role is still unknown; a possible explanation of its role is that high viral load may cause its depletion because calcium ions are involved in viral life processes, such as regulating virus cell entry, gene expression, and virion formation [99]. 

## 4. Role of the Gut Microbiome and Its Metabolites in Health and Disease

### 4.1. Human and Gut Microbiome

The human microbiome is a collection of microbial genomes (consisting of bacteria, bacteriophages, fungi, protozoa, and viruses) living in and around the human body. The Human Microbiome Project (HMP) made it possible to characterize microbial communities across the human body, harboring these microbial cells’ trillions [100]. The human body and microbial undergo a symbiont dynamic to cut conflicts and maximize benefits to the microbiota and the human host, playing essential roles in human health [101]. 

The gastrointestinal microbiome is the most studied due to its relationship with health and abundance in the human body. This complex and dynamic ecosystem includes different microbial communities and is one of Earth’s most populated microbial communities [102].

The colon is the most densely populated microbial in the gastrointestinal tract; meanwhile, only a few species of bacteria are present in the stomach and small digestive tract. There are more than 1500 species comprising 50 phyla [103]. Despite a high degree of interindividual variability in species that varies in abundance and diversity across the digestive tract, the GM has a microbial core microbiome dominated by taxa derived from *Bacteroidetes* and *Firmicutes*, followed by *Proteobacteria*, *Fusobacteria*, *Tenericutes*, *Actinobacteria*, and *Verrucomicrobia*, comprising 90% of the total microbial population in humans [103,104].

The microbiome can be resident and transient; resident microbiota consists of persistent and long-staying microbes, while the transient microbiota consists of temporal and variable harboring time microbes; several factors influence both microbiotas composition, such as environment, diet, age, antibiotic use, and host genetics [105].

The bacterial species in the gastrointestinal tract have a particular function for human health. They influence digestion, metabolism, the immune system, intestinal epithelium’s barrier functions, and antibacterial chemical synthesis. In nutrition, the microbiome influences food energy balance and partitioning, fiber digestion, vitamin and mineral synthesis, and bile acid metabolism [106]. 

The GM develops alongside the immune system and matures during the first few years of life. This bacterial influence is essential for innate immunity (a nonspecific form of immunity) and adaptive immunity (a specific response to an invading pathogen) [107]. 

Some tissues related to microbiota modulation are the spleen, thymus, and lymph nodes. Also, macrophage phagocytosis changes its activity depending on microbiota modulation [82]. Additionally, specific bacteria have a modulatory effect on T cell development as Th1 and Th2 cells, mediated by Type 2 receptor/Type 1 receptor (TLR2/TLR1) heterodimer on lamina propria dendritic cells [108].

### 4.2. Gut Microbiota-Derived Metabolites and Their Function in the Human Host

The GM primarily interacts with the host through gut-derived metabolites, which are small molecules that result from microbial metabolism of dietary substrates and the modification of host molecules such as bile acids (BA) or bacteria, which play an essential role in influencing immune maturation, homeostasis, host energy metabolism, and maintenance of mucosal integrity of the human body [109,110]. Those metabolites have a back-and-forth relationship in maintaining energy and metabolism homeostasis. On one side, gut bacteria profit from the nutrients absorbed by the host; meanwhile, the host relies on gut bacteria metabolites as a substrate for ATP production in the colon [111]. Finally, bacterial metabolites drain from the gut and into the circulation, interfering with the host’s cellular mechanism. Specifically, signals from microbial metabolites influence immune maturation, homeostasis, host energy metabolism, and maintenance of mucosal integrity [112]. Gut-derived metabolites can be broadly classified into three categories: those produced by gut bacteria from dietary sources, those produced by the host and modified by GM, and those generated de novo by gut bacteria (such as polysaccharide A) [113]. At present, it is relevant to understand how metabolites derived from GM are essential regulators of processing and absorbing several nutrients and metabolites such asBA, lipids, amino acids, vitamins, and short-chain fatty acids (SCFAs) [112]. Table 3 mentions how GM-derived metabolites affect host function, which bacterial species are related to, and the association with diseases.

**Figure 2:**
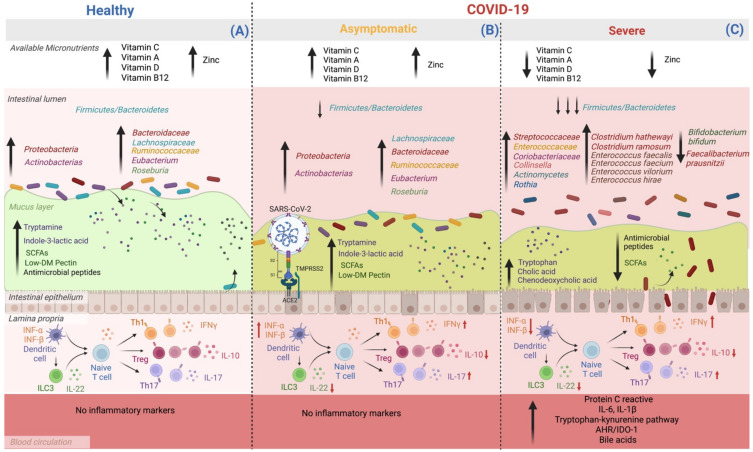
Micronutrient availability, gut microbiota, and its derived metabolites modifications according to COVID-19 severity. Patients with severe complications from COVID-19 have lower availability of micronutrients such as vitamin C, A, D, B12, and Zn compared to asymptomatic and healthy subjects. On the other hand, the dysbiosis of GM seems to worsen as the complications of the disease progress. (**A**) Healthy subjects have an adequate balance in the *Firmicutes: Bacteroidetes* ratio, as well as an abundance of beneficial species belonging to the *Proteobacteria* and *Actinobacteria* phyla, such as *Lachnospiraceae*, *Bacteroidaceae*, *Ruminococcaceae*, *Eubacterium*, and *Roseburia*. Therefore, there is an adequate production of metabolites from the processing of host nutrients: SCFAs, secondary BA, tryptamine, and indole-3-lactic acid. Additionally, some molecules that regulate immune cell function are present, including the balance between Th cell subtypes and their role in controlling a tolerogenic environment and the intestinal barrier. (**B**) In asymptomatic people with COVID-19, no micronutrient deficiency is observed; in addition, although there is an alteration in the GM when compared to uninfected individuals, it is not as marked; the *Firmicutes–Bacteroidetes* ratio decreases, mainly affecting immunological regulation since there is a decrease in the production of IL-22 and IL-10. Also, an increase in IFNγ IL-17, the latter cytokines are necessary for the antiviral response. (**C**) In patients with severe COVID-19, there is a deficiency of vitamins C, A, D, B12, and Zn. In addition, the ratio of *Firmicutes–Bacteroidetes* decreases significantly, the abundance of pathogenic bacteria increases, and those beneficial in pathways decrease nutrient metabolism, which reduces beneficial host metabolites synthesis, such as SCFAs and tryptophan-derived metabolites synthesized by bacteria, such as tryptamine and indole-3-lactic acid. This set of events, together with the intrinsic failure of intestinal transit, also causes the accumulation of primary BA in the colon and an increase in the circulation of primary and secondary BA. Therefore, it increases the damage to the intestinal barrier and bacterial translocation, which leads to a proinflammatory environment with high serum levels of C-reactive protein, IL-6 and IL-1β, proinflammatory cytokines that promote the continuous activation of AHR-IDO-1 and reduces the antiviral response of INF type 1 (α and β) (created by BioRender.com).

SCFAs, including formate, acetate, propionate, butyrate, isobutyrate, valerate, and isovalerate 2-methyl butyrate, hexanoate, and heptanoate, are formed via dietary fermentation in the mammalian gut. Gram-positive anaerobic bacteria, such as *Faecalibacterium*, *Eubacterium*, and *Roseburia* species, degrade amino acids valine, leucine, and isoleucine, generating branched-chain fatty acids and producing butyrate. Propionate may be produced from carbohydrate fermentation either by succinate or acrylate pathway by *Bacteroides*, *Prevotella*, *Alistipes*, *Ruminococcus*, *Phascolarctobacterium*, *Dialister*, *Akkermansia* species. Meanwhile, a wide range of gut microbes, for example, *Bacteroides*, *Bifidobacterium*, *Clostridium*, and *Ruminococcus* species, produce acetate by pyruvate after the release of CO_2_ [114,115,116,117,118,119,120,121,122,123,124,125,126,127,128]. Primary BA are amphipathic molecules from cholesterol stored in the liver. Once there is food intake, BA can be released into the small intestine facilitating the dissolution and absorption of dietary fats, lipids, and lipophilic vitamins. Additionally, BA plays an essential role in the innate immune system by regulating energy regulation and inflammation [129]. Bidirectional interactions between BA synthesis and GM are close and complementary. BA controls gut bacteria overgrowth and protects against inflammation. Meanwhile, the GM helps in the biotransformation of BA through deconjugation, dehydroxylation, and reconjugation processes [130,131,132,133,134,135]. Lipids are organic biomolecules produced and maintained in homeostasis by mammal organisms, dietary fat intake, and bacterial origins in GM [136]. There are eight categories of lipids: fatty acyls, glycerolipids, glycerophospholipids, sphingolipids, sterol lipids, prenol lipids, saccharolipids, and polyketides [136]. Imbalance lipids affect the GM as substrates for bacterial metabolic processes by inhibiting bacterial growth by toxic influence, increasing serum endotoxin load, and exacerbating a severe nonspecific inflammatory response, leading to fat accumulation and redistribution of nutrients in the host defense during the acute phase [137,138,139]. Amino acids are the essential elements of proteins and peptides that regulate signaling pathways and metabolism. GM produces amino acids, including de novo biosynthesis, accessible to the host to balance some amino acid deficiencies in low-quality protein intake [140,141]. Additionally, GM ferment protein and amino acids; the *Clostridium* genus is a fundamental bacteria for lysine or proline utilization, whereas bacteria of the *Peptostreptococcus* genus are the critical driver of glutamate or tryptophan use. Other important bacteria of the genera are *Fusobacterium*, *Bacteroides*, and *Veillonella*, and the species *Megasphaera elsdenii* and *Selenomonas ruminantium* [142,143,144,145,146,147,148,149,150,151,152,153,154]. GM is also capable of producing essential vitamins for human health, including vitamins B2 and B7. Phylas such as *Bacteroidetes*, *Fusobacteria*, and *Proteobacteria* had the potential to produce both vitamins. In the case of vitamin B12, all the *Fusobacteria* examined were found to be producers [143,155,156,157]. Also can modulate neurotransmitters, resulting in behavioral, neurodegenerative, cerebrovascular, and neuroimmune changes. There have been described pathways as “gut–brain-axis” that produce or consume a wide range of mammalian neurotransmitters, including dopamine, norepinephrine, serotonin, or gamma-aminobutyric acid (GABA) [11,140,158,159,160,161,162,163,164,165,166]. Gases are other GM metabolites, such as CO_2_, H_2_, and H_2_S. Although they are not as abundant as other metabolites, they play an important role in human mammal organisms’ functions [11,167,168,169,170,171,172,173,174,175,176,177,178]. Figure 2 focuses on the bioavailability of vitamins, minerals, and microbiota metabolites. The compositional changes highlight three states: individuals without COVID-19, individuals with asymptomatic COVID-19, and patients with severe COVID-19 form.

**Table 3:** Gut microbiota-derived metabolites and their effect on human health and disease.

Group	Metabolite	Species	Target	Function or Effect	Related Disease	References
Bile acids	Cholate, hyocholate, deoxycholate, taurohyocholate, ursodeoxycholate, taurocholate, tauro-α-muricholate, glycocholate, hyodeoxycholate, tauro-β-muricholate, lithocholate, taurodeoxylcholate	*Bifidobacterium*, *Clostridium,* and *Escherichia coli*	farnesoid X receptor (FXR), vitamin D receptor (VDR), steroid and xenobiotic receptor (SXR), constitutive androstane receptor, The Bile Acid Membrane Receptor (TGR5), sphingosine 1-phosphate receptor 2, formyl-peptide receptor, muscarinic acetylcholine receptor	GI mobility and gut permeability, facilitate lipid and vitamin absorption, regulation of GM composition, gut hormones, intestinal immunity, intestinal electrolyte and fluid balance, gut motility, lipid homeostasis, glucose homeostasis, amino acid homeostasis, circadian clocks; influence neurotransmission and physiology	Primary biliary cholangitis, primary sclerosing cholangitis, obesity, nonalcoholic fatty liver disease, non-alcoholic steatohepatitis, atherosclerosis, ulcerative colitis, cancer, hepatic encephalopathy, multiple sclerosis, Alzheimer’s disease, Parkinson’s disease, traumatic brain injury, stroke, and amyotrophic lateral, Inflammatory Bowel Disease	[130,131,132,133,135,179,180]
Gases	H_2_S, H_2_, CO2, CHA, NO	*Desulfovibrio piger*, *Desulfovibrio desulfuricans*, *Escherichia coli*, *Enterobacter aerogenes*, *Enterobacter cloacae*, *Citrobacter freundii*, *Proteus vulgaris*, *Edwardsiella tarda*, *Lactobacillus acidophilus*, *Lactobacillus shirota*, *Lactobacillus rhamnosus*, *Bifidobacterium bifidus*, *Bifidobacterium breve*, *Bifidobacterium infantis*, *Bacillus subtilis*, *Bacillus anthracis*, *Deinococcus radiodurans*	Guanylate cyclase	Slows gut motility, regulates gut inflammation, promotes epitelial secretion and susceptibility to infections, mediation of gastric mucosal, protection and regulate mucosal blood flow	Parkinson’s disease, colitis, ulcer	[11,167,168,169,170,171,172,173,174,175,176,177,178]
Lipids	Lipopolysaccharides (LPS), Conjugated fatty acids, Cholesterol, Phosphatidylcholines, Triglycerides,		LPS targets directly Toll-like receptor 4 (TLR4)	Triggering systemic inflammation, regulation of hyperinsulinemia, immune system, lipoprotein profiles, material bases for bile acid synthesis.	T2D, obesity, nonalcoholic fatty liver disease, hyperinsulinemia, hypercholesterolemia, chronic hepatitis C.	[179]
Neurotransmitters and choline metabolites	GABA, Dopamine, Serotonin and Catecholamines, Methylamine, Dimethylglycine, Dimethylamine	*Escherichia coli*, *Klebsiella pneumoniae*, *Pseudomonas aeruginosa*, *Shigella sonnei and Staphylococcus aureus*, *Lactobacillus brevis and Bifidobacterium dentium* *Anaerococcus hydrogenalis*, *Clostridium asparagiforme*, *Clostridium hathewayi*, *Clostridium sporogenes*, *Desulfitobacterium hafniense*, *Escherichia fergusonii*, *Proteus penneri*, *Providencia rettgeri*, *Providencia alcalifaciens*, *Providencia rustigianii*, *Edwardsiella tarda*, *Yokenella regensburgei*, *Citrobacter freundii*, *Escherichia coli*, *Proteus vulgaris*	Adrenergic receptors, Serotonine (5-HT) receptors, gamma-aminobutyric acid (GABA) receptors Activate NF-KB, protein kinase C, NLR family pyrin domain containing 3 (NLRP3), and inflammasome	Visceral pain, inflammation, and visceral hypersensitivity Inflammation, visceral pain, GI mobility, and psychological factors Changes in Enteric nervous system ENS and gut–brain axis, visceral pain, and visceral hypersensitivity “Regulate gut motility, memory and stress responses, immune function of nervous system” Inhibits bile acid synthesis, promote inflammation, thrombosis, affects myocardial hypertrophy and fibrosis, exacerbates mitochondrial dysfunction	Parkinson’s disease, autism Nonalcoholic fatty liver disease, obesity, aterosclerosis, T2D, heart failure, HBP, Aterosclerosis, Fatty Liver	[100,101,102,103,104]
Others	Ethanol, Methane, Triphosadenine, Lantibiotic, Microcin, Organic acids, Polyamines, Hypoxanthine	*Methaninobrevibacter mithii*, *Methanosphaera stadtmanae*, *Lachnospiracea strains*	Triphosadenine activate Purigenic (P2X and P2Y) receptors	Enhance or damage gut barrier, regulate intestinal or systemic immune response, act as antibiotics to modulate GM composition, supply the nutrients, be toxic to host cells, exacerbating obesity manifestations	Fatty liver disease, C. difficile and H. pylori infections, irritable bowel syndrome, ulcerative colitis, obesity	[179,180,181]
Short-chain fatty acids	Acetate, propionate, butyrate, hexanoate, isovalerate, isobutyrate, 2-methylpropionate, valerate	*Akkermansia muciniphila*, *Alistipes putredinis*, *Anaerostipes hadrus*, *Bacteroides fragilis*, *Bacteroides ovatus*, *Bacteroides uniformis*, *Bacteroides vulgates*, *Bifidobacterium adolescentis*, *Bifidobacterium longum*, *Blautia obeum*, *Clostridium bifermentans*, *Clostridium perfringens*, *Coprococcus catus*, *Coprococcus comes*, *Dialister invisus*, *Eubacterium hallii*, *Eubacterium rectale*, *Faecalibacterium prausnitzii*, *Megasphaera elsdenii*, *Phascolarctobacterium succinatutens*, *Prevotella copri*, *Roseburia hominis*, *Roseburia intestinalis*, *Roseburia inulinivorans*, *Ruminococcus bromii*, *Ruminococcus gnavus*, *Ruminococcus lavefaciens*	G protein-coupled receptors (GPR41, GPR43, GPR109A, GPR81, GPR91) and Histone deacetylases (HDAC1 and HDAC3)	Visceral hypersensitivity and inflammation, regulation of GM composition, gut barrier integrity, appetite, energy homeostasis, gut hormone production, circadian clocks, inhibit proinflammatory cytokines, stimulate water and sodium absorption, modulate systemic immune response	Obesity, T2D, pancreatitis, nonalcoholic fatty liver disease, HBP, atherosclerosis, chronic kidney disease, ulcerative colitis, radiation proctitis, Crohn’s disease, colorectal cancer, autism spectrum disorder, sclerosis, Parkinson’s disease, asthma, diarrhea, hepatocellular carcinoma insulin Resistance	[114,128,179,180]
Tryptophan and indole derivatives	Indole, Imidazole propionate, Indole propionic acid, Indole acetamide, Metyl-indole, Indole acetic acid, Indole lactic acid, Indole pyruvic acid, indoxyl sulfuric acid, Indole aldehyde, Indole-acrylic acid, Indole carboxaldehyde and Tryptamine	*Bacteroides ovatus*, *Bacteroides Adolescentis*, *Bacteroides fragilis*, *Bacteroides pseudolongum*, *Bacteroides thetaiotaomicron*, *Bacteroides eggerthii*, *Bifidobacterium adolescentis*, *Bifidobacterium pseudolongum*, *Burkholderia pvrrocinia*, *Burkholderia Thetaiotaomicron*, *Butyrivibrio fibrisolvens*, *Clostridium botulinum*, *Clostridium caloritolerans*, *Clostridium paraputrificum*, *Clostridium sporogenes*, *Clostridium bartlettii*, *Clostridium bifermentans*, *Clostridium cadaveris*, *Clostridium difficile*, *Clostridium lentoputrescens*, *Clostridium limosum*, *Clostridium lituseburense*, *Clostridium melanomenatum*, *Clostridium paraputrificum*, *Clostridium saccharolyticum*, *Clostridium sporogenes*, *Clostridium tetani*, *Enterobacter cloacae*, *Escherichia Albertii*, *Escherichia coli*, *Eubacterium cylindroides*, *Fusobacterium nucleatum*, *Lactobacillus acidophillus*, *Lactobacillus johnsoni*, *Lactobacillus murinus*, *Lactobacillus reuteri*, *Parabacteroides distasonis*, *Peptostreptococcus anaerobiusm Peptostreptococcus asscharolyticus*, *Peptostreptococcus russelli*, *Rauschbrand bacillus*, *Ruminococcus gnavus*	aryl hydrocarbon receptor (AhR) and PXR	Influence on gut permeability, promotes spore formation, drug resistance, biofilm formation, and virulence; regulate intestinal barrier functions, gut hormone secretion, gut motility, systemic immune response inducing inflammation	T2D, Ulcerative colitis, Crohn’s disease, obesity, stroke, mucosal candidiasis, autism spectrum disorder, Alzheimer’s disease, Parkinson’s disease, migraine, schizophrenia, irritable bowel syndrome, chronic kidney disease, hepatitis, impaired liver function	[142,143,144,145,146,147,148,149,150,151,152,153]
Vitamins	Vitamin D, Vitamin B2. Vitamin, B3, Vitamin B5, Vitamin B6, Vitamin B9, Vitamin B12, Vitamin K	*Salmonella typhimurium*, *Actinobacteria*, *Bacteroidetes*, *and Proteobacteria phyla*	Vitamin receptors	Inflammation and gut permeability Inflammation “Involved in cellular metabolism: modulate immune function and cell proliferation; supply vitamins for hosts”	Schizophrenia, autism, dementia	[143,155,156]

## 5. Gut Microbiota in the COVID-19 Infection and Post-COVID-19 Syndrome Recovery

After microbiota is in dysbiosis, *Firmicutes* reduce in quantity, while *Alistipes* and *Proteobacteria* increase. This phenomenon also occurs when external pathogen agents such as SARS-CoV-2 gain access to the organism. The proportion of the coexisting phyla remains mostly stable and unique within an individual, but it may get altered when the health state is modified. For example, the microbiome of elder populations is considerably decreased in *Firmicutes* and increased in *Proteobacteria* and *Alistipes* [179]. Siew & Herbert [179], determined that individuals infected by SARS-CoV-2 suffer an increase in opportunistic pathogens, including *Streptococcus* y *Actinomyces*, *Rothia*, and reduced *Bifidobacterium bifidum* and *Faecalibacterium prausnitzii*, that also is characterized by an immunomodulator potential that contributes to the host defense with important anti-inflammatory properties, showed a negative correlation. Also, *Ruminococcaceae*, *Bacteroidaceae*, and *Lachnospiraceae* are associate with better health conditions due to their production of short-chain fatty acids; but are considerably reduced in patients with COVID-19 [180,181,182]; meanwhile, opportunistic pathogens such as *Collinsella*, *Staphylococcaceae*, *Enterococcaceae*, and *Coriobacteriaceae* are found in the gut microbiota of critical COVID-19 patients. This overwhelming quantity of microorganisms could be a factor that affects the clinical state of patients due to their intrinsic resistance to several antibiotics and their quick adaptability to chemotherapy [181,183]. Patients in a critical state by COVID-19 admitted into the ICU with concomitant bloodstream infection show an increase in *Enterococcus* and reduced microbial diversity, especially *Bacteroides*, *Ruminococcaceae* and *Lachnospiraceae* [181,184,185]. Similarly, when ICU-admitted patients were compared among COVID-19+ and COVID-19–, *Enterococcus* was overrepresented in COVID-19+ patients; but COVID-19– individuals possessed *Enterobacteriaceae* and *Klebsiella* primarily, as *Enterococcus* was mostly absent [181,182]. Several studies show the relationship between GM composition and COVID-19 symptoms severity. Chakraborty et al. [186,187,188,189] analyzed the composition of this microbiota in 100 hospitalized patients while trying to understand the severity of COVID-19. Findings were: reduced bifidobacterial and commensal microbiota; increased levels of C reactive protein (CRP) and inflammatory cytokines, aspartate aminotransferase, lactate dehydrogenase, and γ-glutamyl transferase. Likewise, there is evidence of enhancement of *Clostridium ramosum*, *Coprobacillus*, and *Clostridium hathewayi*, microorganisms directly related to the severity of disease, *while Faecalibacterium prausnitzii* is inversely correlated; meanwhile, the intestinal virome correlates inversely to COVID-19 severity [190].

The gut microbiome is involved in several biosynthetic pathways, including the biosynthesis of amino acids and nucleotides de novo, carbohydrates metabolism, and glycolysis. The COVID-19-derived dysbiosis shows functional changes due to the disruption of the microorganisms’ ecosystem. Feces samples of COVID-19 patients with mild symptoms and healthy subjects have a microbiome abundantly represented by *Morganella morganii*, *Collinsella tanakaei*, *Collinsella aerofaciens*, and *Streptococcus infantis*, along with some bacteria that produce short-chain fatty acids such *as Bacteroides stercoris*, *Lachnospiraceae*, *Parabacteroides merdae*, and *Alistipes* [189,191].

A study performed on SARS-CoV-2-infected primates to analyze the 16S rRNA profile and β diversity was able to find significant changes in the metabolism and composition of GM, coupled with differences in the concentrations ofBA, a reduction in short-chain fatty acids, and alterations on the metabolism of lipids [192]. Similarly, another study analyzed the gut microbiome of SARS-CoV-2 patients and observed a deteriorated biosynthesis of short-chain fatty acids and L-isoleucine, implying that these functions are essential factors during the development of pathogenesis [193].

## 6. Gut Microbiota Metabolites in the COVID-19 Infection and Post-COVID-19 Syndrome Recovery

The gut microbiome metabolizes nutrients from the diets and transforms them into metabolites that interact with the host cells either directly or indirectly. Some of the most important metabolites, due to their impact on the host’s health, include tryptophan-derived metabolites, SCFAs, and BA-derived metabolites [194]. Next, we will discuss the role and participation of these metabolites in the context of COVID-19.

### 6.1. L-Tryptophan-Derived Microbiota Metabolites

L-tryptophan is an essential amino acid metabolized through three pathways: The Kynurenine pathway is performed by immunological and epithelial cells; the serotonin pathway occurs in enterochromaffin or enteroendocrine cells; lastly, it may get directly converted by GM [195]. Microbiota transforms L-tryptophan into tryptamine, indole-3-ethanol (IE), indole-3-propionic acid (IPA), indole-3-lactic acid (ILA), indole-3-acetic acid (IAA), 3-methylindole, indol-3-aldehyde (IAld), índole-3-acrilic acid (IA), and 3-indoxyl sulfate (I3S) [196]. Each of these molecules serves as a specific ligand for the host cells.

The relevance of tryptophan-derived metabolites relies on their ability to stimulate the aryl hydrocarbon receptor (AHR), a transcription factor expressed in immune cells. AHR is known as an environmental sensor since it will exert a pro-inflammatory or anti-inflammatory effect according to the presence of the molecules from the diet, microorganisms, or contaminants in the microenvironment [197]. There are no studies of tryptophan-metabolizing bacteria in individuals with post-COVID-19 syndrome, but differences are reported in GM between healthy subjects and patients at different stages of COVID-19 severity. These studies show that SARS-CoV-2 infection disrupts gut microbiome equilibrium, increases the number of opportunistic pathogens, and reduces the proportion of beneficial bacteria [198,199]. Therefore, we can assume that during COVID-19, there may be an alteration in the metabolism of tryptophan. Accordingly, Tan et al. [200] described that the population of indole-producing aerobic bacteria increases during dysbiosis episodes, for example, *E. coli*. This is associated with higher production of the atherosclerotic metabolite 3-indoxyl sulfate, which causes damage to vascular cells and induces the expression of IL-6 upon access into the bloodstream. The bacterial species *Lactobacillus*, *Ruminoccocus*, *Brautia*, *Bifidobacterium,* and Spore-Forming Bacteria are beneficial to the host’s health. Tryptophan-decarboxylase (TrpD), tryptophanase (TnaA), tryptophan monooxygenase, and phenyllactate dehydratase (fldAIBC), enzymes are responsible for the production of tryptamine, IAld, IAA, IPA, and IA, respectively [201]. These metabolites have shown anti-inflammatory effects both in vitro and in vivo; for example, their interaction with AHR stimulates IL-22 synthesis and, as a consequence, the production of antimicrobial peptides; they also enhance the expression of IL-10 in the gut epithelial cells, diminish the release of pro-inflammatory cytokines such as IL-1β and IL-6 from peripheral blood mononuclear cells, and reduce the inflammatory effects of hepatocytes in response to TNF-α [202]. In addition, tryptamine acts as a neurotransmitter, vasoconstrictor, vasodilator, antioxidant, and antibacterial agent [203].

Liu et al. [204] described that in COVID-19 patients, dysbiosis is related to the susceptibility to develop long-term complications. Regarding tryptophan metabolism, the microbiota from patients with sequelae possessed a marked decrease of the bacterial species *Brautia* (responsible for degrading tryptophan into tryptamine) and *Bifidobacterium longum* (responsible for degrading tryptophan into indole-3-lactic acid). A low expression of Solute Carrier Family 6 Member 19 (SLC6A19) has been observed on the surface of the small intestine cells of COVID-19 patients because of its co-internalization along the ACE2, the preferred receptor for SARS-CoV-2. Whenever SARS-CoV-2 meets its receptor, SLC6A19 is internalized as well and thus reduces the absorption of tryptophan by the small intestine epithelial cells [205,206]. The reduced SLC6A19 activity disrupts the activation of mTOR, which leads to lower secretion of antimicrobial peptides from the Paneth cells in the small intestine, thereby altering the equilibrium of the GM and thus increasing the susceptibility towards gut inflammation [207,208]. These data suggest that tryptophan increases in the lumen and bloodstream due to gut dysbiosis and the response of the infected gut cells. Gagandeep Kaur et al. [209] confirm the latter finding a higher serum concentration of tryptophan and its metabolites in COVID-19 patients compared to healthy controls by ultraperformance liquid chromatography-tandem mass spectrometry (UPLC-MS) quantification.

In addition to the epithelial cells, the immune system cells express Indoleamine-2,3-dioxygenase (IDO) 1 in response to pro-inflammatory cytokines [210]. Usually, the goal after activation of IDO/AHR in the gut cells is to metabolize the tryptophan to reduce its availability in the microenvironment and thus affect the survival of bacterial, parasitic, and viral pathogens, thereby controlling acute and chronic infections [211]. However, in the COVID-19 context, the greater availability of tryptophan will allow for its metabolism through the kynurenine pathway, whose metabolites will induce the expression of the pro-inflammatory IL-1β, IL-10, TNF-α, IL-6 by AHR, further increasing the local and systemic inflammation [196,212].

In the groups vulnerable to developing severe COVID-19, such as elderly populations and individuals with the comorbidities Diabetes Mellitus II and Cerebrovascular Disease, the kynurenine in this pathway is highly activated [196,212], which could lead to an increase of pro-inflammatory cytokines and constitutive AHR activation, which is related to the reduced antiviral response by INF-1 [213,214].

### 6.2. SCFAs

SCFAs are biologically active metabolites produced by the GM. Exogenously, they derive from fermenting dietetic fiber [215]; endogenously, to a lesser extent, they are formed by the host through lipid oxidation and metabolism of branched-chain amino acids [216]. Up to 95% of total SCFAs produced in the intestinal lumen are acetate, butyrate, and propionate [217].

Butyrate is the primary energy source for colonocytes; thus, it is an indispensable molecule for their proper functioning [218]. Around 70% of acetate is metabolized in the liver and transformed into an energy source for hepatocytes and a substrate for synthesizing cholesterol, long-chain fatty acids, glutamine, and glutamate. Between 30–50% of propionate is absorbed in the liver, where it serves as a precursor for gluconeogenesis [219,220]. Besides the energetic output, a critical activity attributed to these metabolites is their ability to modulate the immune system. This occurs mainly in specific receptors interaction such as free acid receptor (FFAR) such as FFAR2R (GPR43), FFA3R (GPR41), GPR109A, and Olfactory receptor (Olfr78), which are differentially expressed across the host cells [221]. In addition, they can regulate gene expression through the inhibition of HDAC regardless of GPR receptors [222].

Production of SCFA is proven beneficial, especially regarding immune tolerance and establishing an anti-inflammatory environment [223]. Some mechanisms involved include promoting differentiation of Treg cells stimulated by butyrate, regulation of the FOXP3+ transcription factor, or inhibiting HDAC [224,225]. In addition, they modulate the expression of pro-inflammatory cytokines by intestinal dendritic cells and macrophages [226]. Moreover, the activation of B lymphocytes produces antibodies [227]. Their effects also extend to innate immunity, where they regulate the microbicide mechanisms of neutrophils, which constitute up to 60% of total circulating leukocytes. Moreover, the cell migration of neutrophils, their expression of adhesion molecules, apoptosis, ROS production, and cytokines release [228]. Furthermore, extracellular trap formation will depend on the concentration and proportion of SCFA [229].

The concentration and proportion of SCFAs in the intestinal lumen and bloodstream vary according to diet, GM, and physiological status [230]. As a consequence of COVID-19-derived dysbiosis, SCFAs have been proposed as a support treatment to prevent the cytokine storm and multiorgan failure in COVID-19, especially butyric acid due to its immunoregulatory activities [231].

COVID-19 patients with dysbiosis suffer from a decrease in SCFA-producing bacteria. A cohort performed in two hospitals in Hong Kong (n = 100) identified an important reduction of *F. prausnitzii* and *Bifidobacterium bifidum*, which was negatively correlated to the severity of the disease [188]. Similar results were observed by Lingling Tang et al. [198], who studied COVID-19 patients with pneumonia and discovered an increase in opportunistic bacteria such as *Enterococcus* and *Eneterobacteriacea* coupled with a decrease of butyrate-producing bacteria (BPB) such as *F. prausnitzii* compared to patients with only mild symptoms. The reduction of BPB was correlated to a higher presence of inflammatory markers such as C reactive protein and the number of neutrophils in the group of severe COVID-19.

Ruquin Lin et al. [232] reported last June an article wherein they compared the GM of 81 individuals, including asymptomatic and ambulatory cases, as well as those with adverse results and severe acute respiratory syndrome. They observed that SCFA-producing species were enriched in healthy subjects and asymptomatic COVID-19 patients compared to the other groups. These species also correlated negatively with adverse outcomes. They analyzed the profile of metabolic pathways and observed that in these individuals, the pathways of sucrose were enriched. They also observed that in all groups infected with SARS-CoV-2, the expression of carbohydrate-active enzymes (CAZymes) decreased, suggesting an inadequate degradation of polysaccharides. In addition, in all individuals with COVID-19, except in the asymptomatic group, the abundance of SCFA-producing species decreased markedly.

Fen Zhang did observe similar results in their study population. They found depletion of SCFA-producing bacteria in COVID-19 patients as well as disruption of microbial functional pathways in the more severe states of disease. The Bifidobacterium pathways for acetic acid synthesis were especially affected by the 2.4-fold reduction. In addition, they found negative correlations between the functionality of four pathways involved in the production of SCFAs and the presence of the heath failure marker NT-proBNP, a metabolite previously associated with COVID-19 severity [193].

In order to study whether the dysbiosis would remain in patients after COVID-19, they evaluated the GM and its functions 30 days after hospital discharge. The microbiota of patients with severe disease was still affected and, even though recovery of bacterial species was similar to the reference group, nine pathways involved in the biosynthesis of SCFAs were persistently exhausted 30 days after disease resolution [193].

After identifying that SARS-CoV-2 infection disrupts the GM, in vitro experiments were conducted to study whether the colonocytes were also affected. Lívi Pascoal et al. observed that expression of RIG-I and IFN-β was significantly enhanced in colon biopsies of COVID-19 patients compared to the control group. In addition, the expression *INF-III*, *IFNLR1*, *DDX58*, and *TMPRSS2* was reduced after incubating with SCAFs. However, no differences in SARS-CoV-2 viral load were observed after incubating the cells either with or without SCFAs [233].

The differences in the GM and the production of SCFAs have been well described in the context of COVID-19. However, further clinical and preclinical studies are required to dilucidated the specific mechanisms of the local and systemic effect of SCFAs during infection by SARS-CoV-2 and the recovery from the disease.

### 6.3. Microbial Metabolites Derived from Bile Acids

Lipids are macronutrients that need to get through emulsification and hydrolysis in order to get absorbed, processes that require a group of steroid molecules known as BA [234]. Primary BA are synthesized in the liver from cholesterol, cholic acid, and chenodeoxycholic acid through their conjugation with glycine or taurine. Then they are secreted onto the small intestine where they become transformed by the GM into secondary BA, mainly deoxycholic acid (DCA) and lithocholic acid (LCA) [235].

Secondary BA act as ligands for the nuclear receptors FXR, VDR, and PXR in epithelial and endothelial cells as well as hepatocytes. They also interact with TGR5, a membrane-bound receptor expressed in the gut, pancreas, lymphoid tissue, and brain. Both DCA and LCA are important molecules derived from the GM due to their ability to regulate the immune system through the above-mentioned receptors [221].

The aforementioned study by Ruquin Lin et al. [232] grouped COVID-19 patients according to the severity of disease in a wide range from asymptomatic patients and up to those in the critical stage. They reported that the *Firmicutes/Bacteroidetes* ratio in the GM from patients gets progressively more affected as the severity of disease increases. In addition, the GM from asymptomatic patients preserves the probiotic species *Faecalibacterium prausnitzii*, *Bifidobacterium longum*, *Blautia obeum*, *Roseburia hominis*, and *Ruminococcus*. These bacteria are negatively correlated to the adverse outcomes derived from COVID-19. Furthermore, their microbiota, unlike the critical patients’ microbiota, was characterized by a high expression of genes involved in the metabolic pathways for the biosynthesis of secondary BA. Harry Sokol et al. [192] observed the effect of SARS-CoV-2 infection on primate microbiota and found that the quantity of total BA increased along with disease severity. Notably, the ratio of primary/secondary BA was also acutely higher.

These data suggest that the disruption of the GM by SARS-CoV-2 infection gets worse as the disease advances in severity. As the dysbiosis grade increases, the intrinsic functions of the ileum get further altered, leading to higher intestinal transit that prevents the complete reabsorption of BA, thereby increasing its concentration in the colon. Moreover, the GM in critical COVID-19 patients is functionally limited and thus, the BA gets concentrated in the feces of these patients [236].

The high concentration of BA at the intestinal lumen promotes epithelial cell dysfunction derived from failures at the membrane transporters [236], which leads to mitochondrial stress and cell death. In turn, tight junction proteins, specifically occludin, get downregulated and provoke higher cell and paracellular permeability among enterocytes, increasing the BA concentration in the bloodstream and peripheral tissues [237].

The serum BA profile shows that patients with acute respiratory distress syndrome (ARDS) match these observations [238], so it could be inferred that, in the critical COVID-19 context, the elevated concentration of BA may damage the intestinal barrier and reach peripheral tissues including lungs, heart, kidneys, and endothelium through the bloodstream. Their cytotoxic activity may injure the cell membranes in peripheral tissues, leading to a local and systemic inflammatory response that shows up clinically.

## 7. Conclusions

Accumulated data on the role of micronutrients in response to viral infections and specifically to SARS-CoV-2 demonstrate their role in immune regulation as a crucial therapeutic support tool. Asymptomatic Patients with COVID-19 have greater availability of micronutrients and microbial metabolites than those patients with severe complications.

Whether individuals obtain vitamins and minerals through diet or supplementation, a correct diet as primary care stimulates homeostasis in the micronutrient pool in the individuals as in the GM and its synergic derived metabolites. Research highlights the importance of promoting micronutrients as therapy, coupled with pharmacological treatment for the prevention, severity reduction, and boost recovery from COVID-19 and the post-COVID-19 syndrome. However, clinical studies are lacking to determine the dosage, outcome, and efficacy of use. 

## Figures and Tables

**Table 1:** Function and clinical uses of micronutrients.

Vitamins	Food Sources	Deficiency Symptoms	Risk Factors for Micronutrient Deficiency	Functions and Clinical Uses	References
Water-soluble vitamins					
Thiamin (B1)	Whole grains Pork Fish Yeast	Fatigue Anorexia Beriberi Endocarditis Arrhythmias Sleep disorders Irritability Neuropathy Wernicke–Korsakoff syndrome	Chronic alcoholism Malnutrition Bariatric surgery Pregnancy Breastfeeding Diarrhea Chronic kidney disease	Wernicke–Korsakoff syndrome Carbohydrate metabolism (thiamine pyrophosphate)	[12,13,14,16]
Riboflavin (B2)	Eggs Dairy Green vegetables Meat Mushrooms Almonds	Ariboflavinosis (stomatitis, cheilitis and glossitis) Photophobia Dermatitis Cataracts Migraines Changes in personality	Anorexia nervosa Malabsorption syndromes Prolonged use of barbiturates Pregnancy Dialysis Diarrhea	Migraine prophylaxis Cataract prevention Coenzyme in multiple biochemical processes	[12,13,14]
Niacin (B3)	Animal and plant foods Soy Nuts Seeds Legumes Grains	Pellagra Depression Anxiety Memory loss Psychotic symptoms	Low Tryptophan Intake High Corn Diet Hartnup Disease Carcinoid syndrome	Coenzyme in oxidation-reduction reactions	[12,13,14]
Pantothenic acid (B5)	Fortified cereals Fish Avocado Eggs Beef and pork Sunflower seeds Lentils	Alopecia Dermatitis Numbness Encephalopathy Behavior changes	Deficiency associated with another B complex vitamin	Acne, Allergies Rheumatoid Arthritis	[12,13,14]
Pyridoxine (B6)	Beef Poultry Starchy vegetables Fortified cereal	Anemia Irritability Depression Seizures Peripheral neuropathy	Alcoholism Kidney failure Rheumatoid arthritis Malabsorption syndromes	Nausea and vomiting during pregnancy	[12,13,14]
Biotin (B7)	Meat Eggs Fish Seeds Soy Nuts	Dermatitis and tremor in extremities Depression Irritability Seizures Cognitive impairment	Alcoholism Epilepsy medications Pregnancy Biotinidase enzyme deficiency	Hereditary enzyme deficiency Brittle hair syndrome Fatty acid synthesis Glucose utilization Protein metabolism	[12,13,14]
Folic acid (B9)	Green leafy vegetables Nuts Beans Dairy Meat Poultry Brussels	Megaloblastic anemia Behavioral changes Psychosis Dementia	Genetic polymorphisms Malabsorption Poor intake Hemodialysis Hemolysis	Megaloblastic anemia Prevents neural tube defects in pregnancy Dialysis Malabsorption	[12,13,14,16]
Cobalamin (B12)	Animal foods Fortified foods	Megaloblastic anemia Behavioral changes Psychosis Dementia	Pernicious anemia Malabsorption Vegan diet	Megaloblastic anemia Malabsorption syndromes Maintenance dose for deficiency in vegans Essential in red blood cell production	[12,14,16]
Vitamin C	Oranges Lemons Grapefruit Green vegetables Beef liver	Scurvy	Consumption deficit	Collagen formation Wound healing Immune system Antioxidant Free radical scavenger Skin disorders (Redness, hyperpigmentation)	[12,14,16]
Fat-soluble vitamins					
A	Animal foods Human milk Fish Liver Eggs Green and yellow vegetables Yellow and orange non-citrus fruits	Visual impairment Bitot spots Keratomalacia Follicular hyperkeratosis Growth retardation Respiratory and intestinal infections	Malnutrition Poverty	Maintenance of visual sharpness Growth and development Formation of red blood cells Formation of skin and bones Immunity	[12,14,16]
D	Exposure to sunlight Milk Cheese Fortified cereals Egg yolks Salmon	Osteomalacia Rickets	Decreased exposure to UV rays Absorption disorders	Stimulates bone mineralization Antioxidant Improves the absorption of phosphorus and calcium	[12,13,14,16]
E	Wheat germ Oil nuts Cereal Meat Egg Milk Green leafy vegetables	Cystic fibrosis Ataxia Abetalipoproteinemia Hemolysis Macrocytic anemia in premature infants	Poor food intake	Antioxidant Wound healing Immunity	[12,14,16]
K	Fresh green leafy Vegetables Egg yolk Soybean oil Liver	Generalized bleeding Haemorrhagic disease in the newborn Prolongation of coagulation times	Poor food intake Coagulation factor deficiency	Formation of prothrombin and other K-dependent coagulation factors	[12,14,16]
Minerals					
Zn	Red meat Fish Poultry Nuts Whole grains	Gastrointestinal diseases Decreased immune function	Alcoholism Chronic Kidney disease	Cell-mediated immunity Bone formation Tissue growth Brain functions Sexual maturation Fertility	[12,14]
Calcium	Seafood (salmon and sardines) Green leafy vegetables Milk Egg	Fractures Osteoporosis Osteomalacia Rickets	Alterations in parathormone and calcitonin Chronic kidney disease Alcoholism Magnesium deficiency	Bone growth and development Nerve function Muscle contraction Blood clotting	[13,14]

**Table 2:** Role of micronutrients in immunomodulation and their deficiency effects on immune system.

Nutrient	Immunomodulating Property	Deficiency Effect on Immune System	References
Vitamin A	It maintains the integrity of the barrier and the normal differentiation of epithelial tissues Mucosal immune response and anti-inflammatory agent It regulates the functions of NK cells and the activities of macrophages Differentiation and development of T helper 1 (Th1) and T helper 2 (Th2) cells Supports the production of antibodies by B cells	Increases susceptibility to virus-induced infections of the respiratory tract, measles, and diarrhea Failure of immune responses to vaccines	[17,18,19]
Vitamin C	It contributes to the maintenance of the redox integrity of cells and protection against (ROS) generated by inflammatory responses Regenerates other antioxidants It stimulates the functions of leukocytes It contributes to the integrity of the epithelial barrier by promoting collagen synthesis Antimicrobial Activities: Increases serum complement protein and IFN γ production Role in antibody production	Increases the risk and severity of some respiratory infections, including pneumonia	[17,18,19,20,21]
Vitamin D	Production of antimicrobial peptides (catelicidin and defensin) responsible for modifying the GM Promotion of anti-inflammatory cytokines Inhibition of IFN γ nuclear factor kB It improves innate immunity by increasing the differentiation of monocytes to macrophages Promotes the growth and phagocytic capacity of macrophages	They increase the risks, severity, and mortality of various respiratory conditions, such as rhinitis, asthma, tuberculosis, chronic lung disorders, viral respiratory infections, including COVID-19	[17,18,19,21]
Vitamin E	Lipid-soluble antioxidant that protects cell membranes against oxidative damage Supports the integrity of airways and epithelial barriers Enhances the cytotoxic activity of Nκ cells It modulates the expression of IFN γ and Interleukin 2 It decreases the expression of prostaglandin E2 by macrophages Optimizes and improves Th1 function	It impairs the functions of humoral and cellular adaptive immunity, thus facilitating viral infection with highly virulent strains, and conditions serious subsequent pathologies together with abnormal immune responses	[17,18,19]
B6	Participates in biosynthesis of fatty acids and proteins along with B12 and B9 Maintains Th1 response Involved in the proliferation of T lymphocytes	The deficiency is accompanied by suppression of the Th1 response and promotion of Th2 as well as a decrease in pro-inflammatory cytokines	[17,19]
B12	Participates in biosynthesis of fatty acids and proteins along with B6 and B9 Effects on cytotoxic cells (NK, CD+, T cells)	Suppresses NK cell activity, decreases the number of lymphocytes and abnormal CD4+/CD8+ cell ratio	[17,19]
Folic acid	Participates in biosynthesis of fatty acids and proteins along with B12 and B9 Maintains innate immunity (NK cell activity)	It causes an impaired immune response and resistance to infection Increased carcinogenicity due to reduced cytotoxic activity	[17,19]
Magnesium	Involved in nucleic acid metabolism, DNA replication, leukocyte activation, regulation of apoptosis Protects DNA from oxidative damage	Increases susceptibility to upper respiratory tract infections It promotes low-grade chronic inflammation through the production of pro-inflammatory cytokines, acute phase proteins, and free radicals.	[17,19]
Se	Key role in redox and antioxidant regulation through glutathione peroxidases by scavenging free radicals Essential for the optimal immune response through the regulation of IFN α, IFN γ, and IFN β production Participates in the production of Immunoglobulin G Influences the functions and differentiation of NK cells, T cells, and antibodies	Increases the risk and virulence of viruses including lung infections, particularly in infants, during their first six weeks of life	[17,18,21]
Zn	It modulates the function of approximately 2000 enzymes and 750 transcription factors, which include immune, growth, and development processes Antiviral properties: Inhibits the enzyme RNA polymerase Maintains the integrity of the immune barrier Improves the cytotoxic activity of NK cells Participates in complement protein activities and IFN γ production It intervenes in the cytotoxic defense against oxidative stress	It increases the risk and morbidity of inflammatory disorders, infections, and viral pneumonia, particularly in children and the elderly Increases the risk of bacterial and fungal infections (particularly diarrhea and pneumonia)	[17,18,19,20]
Fe	Essential for cell differentiation and growth. Involved in DNA synthesis Involved in the process of destruction of bacteria by neutrophils through the formation of toxic hydroxyl radicals	It decreases the secretion of cytokines (IFN γ, TNFα, Interleukin 2 [IL-2]) It attenuates the activity of NK cells and lymphocytes	[17,19]
Cu	Maintains intracellular antioxidant balance Important role in innate immunity (macrophages, neutrophils, and monocytes)	It decreases the proliferation of T cells and increases the circulation of B cells There are no reports of increased incidence of infections during decreased intake	[17,19]
